# Comprehensive Analysis of Inhibitor of Apoptosis Protein Expression and Prognostic Significance in Non–Small Cell Lung Cancer

**DOI:** 10.3389/fgene.2021.764270

**Published:** 2021-12-02

**Authors:** Jun Liu, Yi Lu, Wenan Huang, Zhibo He

**Affiliations:** ^1^ Medical College, Jiujiang University, Jiujiang, China; ^2^ School of Literature and Communication, Jiujiang University, Jiujiang, China

**Keywords:** IAPS, LUAD, LUSC, diagnose biomarker, clinical stages, prognostic values, correlationship

## Abstract

Inhibitors of apoptosis proteins (IAPs) have been associated with tumor development and progression by affecting apoptosis through cell death signaling pathways. To date, eight IAPs (BIRC1–8) have been identified in mammalian cells. However, the role of IAPs in non–small cell lung cancer (NSCLC) development and progression has not been explored in depth. In this study, we used public datasets and bioinformatics tools to compare the expression, prognostic significance, and function of IAPs in NSCLC and its subtypes. Expression of IAPs in cancer and normal tissues and at different stages of NSCLC was compared with gene expression profiling interactive analysis, and their prognostic significance was analyzed with the Kaplan–Meier Plotter database. The correlations among IAPs were analyzed with the STRING database and SPSS19.0. Functional annotation of IAPs was analyzed by Gene Ontology and Kyoto Encyclopedia of Genes and Genomes enrichment on the basis of the DAVID tool. Among patients with lung adenocarcinoma (LUAD), the expression level of *BIRC5* was higher than that in normal samples, and the expression of *BIRC1* and *BIRC5* significantly varied in different stages. Moreover, the *BIRC1*–*3* and *BIRC5* mRNA levels were associated with overall survival (OS), and the *BIRC1*–*2* and *BIRC5*–*6* mRNA levels were associated with progression-free survival (PFS). Among patients with lung squamous cell carcinoma (LUSC), the expression level of *BIRC1* was lower and that of *BIRC5* was higher than those in normal tissues, and *BIRC5* expression significantly varied in different stages. *BIRC1* expression was associated with OS, whereas *BIRC2* and *BIRC6* expression was associated with PFS. Enrichment analysis showed that most IAPs are associated with ubiquitin- and apoptosis-related pathways. Collectively, this study suggests *BIRC5* as a potential diagnostic and staging marker, *BIRC1* as a potential marker of OS, and *BIRC2* and *BIRC6* as potential PFS markers for patients with NSCLC. These highlight new targets for the early detection, treatment, and management of NSCLC.

## Introduction

Non–small cell lung cancer (NSCLC) has one of the highest mortality rates among malignant tumors globally, which accounts for approximately 80% of all lung cancers (Siegel et al., 2020). The two predominant histological phenotypes of NSCLC are lung adenocarcinoma (LUAD, ~50% of cases) and lung squamous cell carcinoma (LUSC, ~40% of cases) (Davidson et al., 2013; Langer et al., 2015). Unfortunately, currently, available biomarkers mainly reflect sex and age variations (Tsao et al., 2012) but cannot accurately identify the stage or prognosis of a tumor. Consequently, NSCLC remains difficult to detect at an early stage, and most patients are commonly diagnosed when the cancer has already progressed to an advanced stage (40% of NSCLC cases are diagnosed at stage IV) and thus not eligible for curative treatments. Therefore, the prognosis of NSCLC remains poor with a 5-year survival rate of only 2%–13% (Liu et al., 2020). Currently, the primary treatment of NSCLC is surgery, radiotherapy, and chemotherapy (Upadhya et al., 2021). Because of tumor heterogeneity, the current biomarkers used to predict NSCLC prognosis have some limitations; thus, it is necessary to explore new biomarkers as diagnostic and prognostic indicators to effectively improve survival and individualized treatment.

Inhibitors of apoptosis proteins (IAPs) are among the most extensively studied molecular and therapeutic targets in treating cancers, and their dysregulated expression has been reported in NSCLC (De-Xuan et al., 2017; Mazur et al., 2018). IAPs play essential roles in preventing apoptosis or programmed cell death. To date, eight IAPs have been identified in mammalian cells (BIRC1–8; see Table 1). The common feature of IAP family members is the presence of one or more baculoviral IAP repeats (Kumar et al., 2020). In addition to inhibiting apoptosis, IAPs play various biological roles, including regulation of innate immunity and inflammation, cell proliferation, cell migration, and apoptosis (Ji et al., 2018; Khan et al., 2021). Accordingly, IAPs act as pivotal regulators in oncogenesis by directly or indirectly affecting apoptosis through intrinsic and extrinsic cell death signaling pathways (Ji et al., 2018; Khan et al., 2021). Therefore, dysregulation of IAPs may lead cells toward cancerization (Yang and Wang, 2016; Yang et al., 2020; Zhang et al., 2021).

**TABLE 1:** Inhibitor of apoptosis proteins information.

No	Gene symble	Gene ID	Also known as
1	NAIP	4,671	**BIRC1**; NLRB1; psiNAIP
2	BIRC2	329	API1; MIHB; HIAP2; RNF48; cIAP1; Hiap-2; c-IAP1
3	BIRC3	330	API2; MIHC; CIAP2; HAIP1; HIAP1; IAP-1; MALT2; RNF49; c-IAP2
4	XIAP	331	API3; ILP1; MIHA; XLP2; **BIRC4**; IAP-3; hIAP3; hIAP-3
5	BIRC5	332	API4; EPR-1
6	BIRC6	57,448	APOLLON; BRUCE
7	BIRC7	79,444	KIAP, LIVIN; ML-IAP; MLIAP; RNF50
8	BIRC8	112,401	ILP-2; ILP2; RNF136; hILP2

Downregulating *BIRC2* expression indirectly induces NSCLC cell apoptosis by preventing the formation of the caspase-8–activating platform (Yang and Wang, 2016; Jian et al., 2019). Moreover, the positive rates of *BIRC4* mRNA expression in pathological tissues of patients with NSCLC were reported to be significantly higher than those in the para-cancerous tissues (De-Xuan et al., 2017). *BIRC5* is strongly expressed in different types of tumors but is not expressed or is only expressed at low levels in most normal differentiated tissues (Xiao and Li, 2015; Mazur et al., 2018). These findings suggest a role of IAPs in NSCLC. However, the underlying mechanism and functions of IAPs in different subtypes of NSCLC or at different stages of cancer progression have yet to be fully elucidated.

RNA and DNA research, an essential component of biological and biomedical studies, has been revolutionized with the development of microarray technology, providing vast molecular data for comparative analysis. However, to the best of our knowledge, bioinformatics analysis of IAPs has yet to be applied for NSCLC. In this study, we comprehensively analyzed the expression of IAPs in patients with NSCLC using public datasets to determine their expression patterns, potential functions, and distinct prognostic value. This study can provide new insight into understanding the molecular mechanisms of IAPs in NSCLC toward development of drugs to inhibit aberrantly expressed IAPs that can help to induce apoptosis in cancerous cells. Moreover, exploring biomarker to diagnose lung cancer and distinguish stages is very necessary.

## Materials and Methods

### Ethics Statement

This study was approved by the Academic Committee of Jiujiang University. All datasets were retrieved from the published literature, in which written informed consent from patients was confirmed for the individual studies.

### IAPs Expression Analysis

The differential mRNA expression of IAPs between NSCLC and normal samples was evaluated separately for LUAD and LUSC with gene expression profiling interactive analysis (GEPIA2; http://gepia2.cancer-pku.cn/#index/). The “expression analysis” mode was selected, with each IAP (*BIRC1 BIRC2*, *BIRC3*, *BIRC4*, *BIRC5*, *BIRC6*, *BIRC7*, and *BIRC8*) added as input. LUAD and LUSC were selected as cancer types. Differentially expressed genes were selected according to a log2 fold change cutoff of 2 and q-value < 0.05. All other options were set to the default values.

### Prognostic Significance of IAPs Expression in NSCLC

To further explore whether IAPs can be potential prognostic biomarkers in NSCLC, we evaluated the prognostic value of *BIRC1*–*7* mRNA expression in the survival of patients with LUAD and LUSC separately using the Kaplan–Meier Plotter database (https://kmplot.com/analysis/); data on *BIRC8* mRNA expression and survival of patients with LUAD and LUSC are lacking from the database. Patient samples were split into two groups according to the median expression level (high versus low expression). The Kaplan–Meier curve, hazard ratio with 95% confidence interval, and log-rank *p*-value were used to evaluate the relationship between the expression of each IAP and the overall survival (OS) or progression-free survival (PFS) of patients with NSCLC (LUAD and LUSC).

### Construction of the IAPs Protein-Protein Interaction Network

The PPI network was constructed from the STRING database (https://string-db.org/), which includes data compiled from several sources. “BIRC1, BIRC2, BIRC3, BIRC4, BIRC5, BIRC6, BIRC7, and BIRC8” were input to the “multiple proteins” box with “*Homo sapien*s” selected as the organism. Other options were left as default options. Cytoscape 3.7.1 software was used for construction of the PPI network and further visualization for analysis.

### Correlations of IAP mRNA Levels in Patients With NSCLC

Gene Expression Omnibus profiles (https://www.ncbi.nlm.nih.gov/geoprofiles/?term=) were used to determine the correlations among expression levels of IAPs in NSCLC, using the keywords “BIRCX NSCLC” (where X refers to 1–8 for the eight IAPs), and each profile was obtained (https://www.ncbi.nlm.nih.gov/geoprofiles/62790008). Scatter plots were constructed and pairwise correlations between all IAPs were analyzed according to the Pearson correlation coefficient using SPSS 19.0 software; *p* < 0.05 was considered statistically significant.

### Functional Enrichment Analysis of IAPs

The biological functions of IAPs were analyzed using Gene Ontology (GO) terms and Kyoto Encyclopedia of Genes and Genomes (KEGG) pathways on the basis of the DAVID tool (https://david.ncifcrf.gov/). GO annotation enrichment analysis was conducted to identify the unique biological properties of IAPs, including biological processes, cellular components, and molecular functions. The top five terms were selected according to the *p* value. KEGG pathway enrichment analysis was performed to explore the key pathways of IAPs; *p* < 0.05 was considered statistically significant.

## Results

### Transcriptional Levels of IAPs Are Altered in NSCLC

**FIGURE 1:**
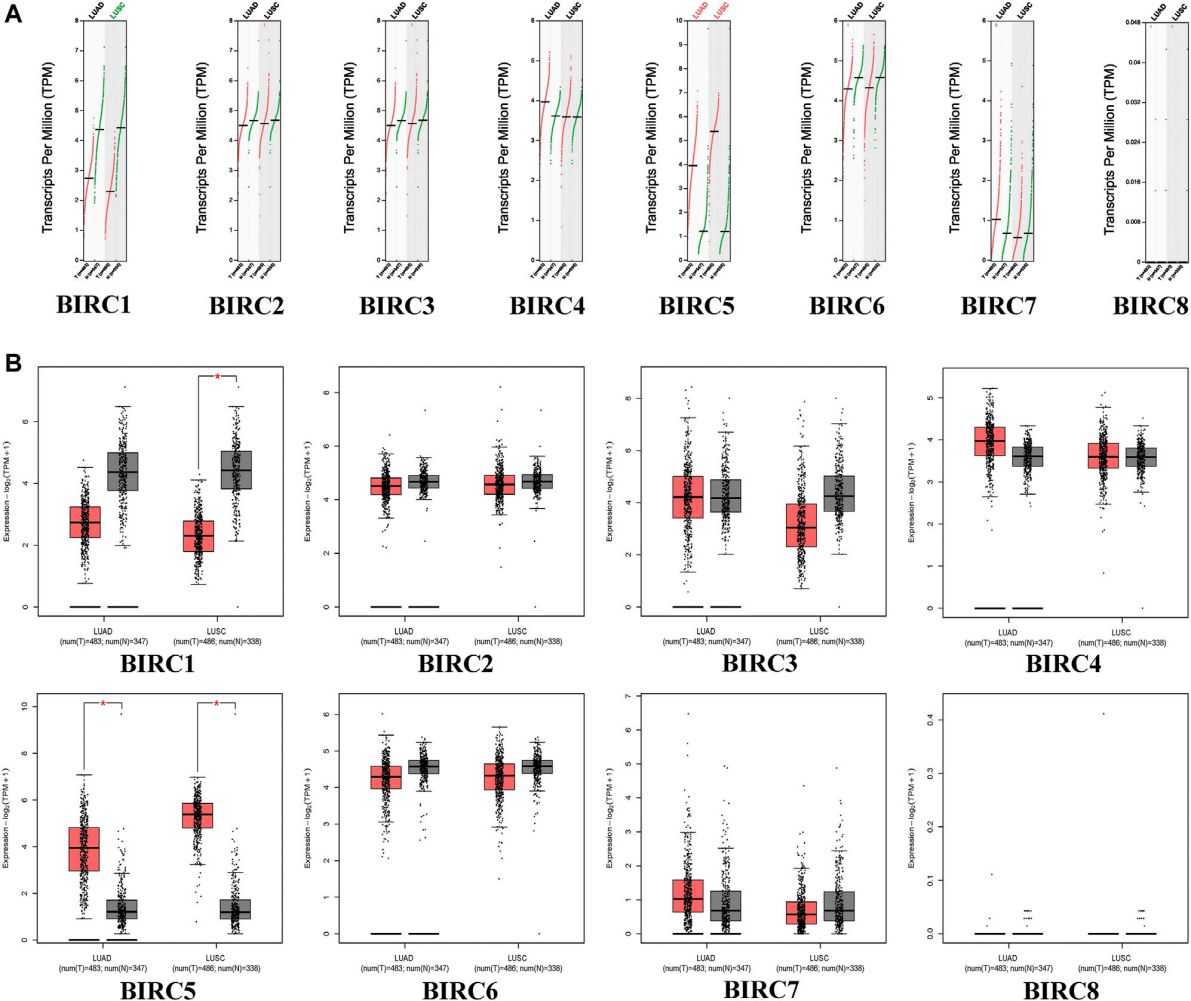
mRNA expression of IAPs between NSCLC and normal lung tissues. **(A)** Scatter diagram of IAPs expression in NSCLC; red dots indicate tumor tissues and green dots indicate normal tissue. Green text indicates that the gene expression level in the tumor tissues was lower than that in normal tissues, and red text indicates that the gene expression level in tumor tissues was higher than that in normal tissues. **(B)** Box plot of IAPs expression in NSCLC; red boxes indicate tumor samples and the gray boxes indicate normal samples. *p < 0.05. NSCLC: non–small cell lung cancer; LUAD: lung adenocarcinoma; LUSC: lung squamous cell carcinoma.

**FIGURE 2:**
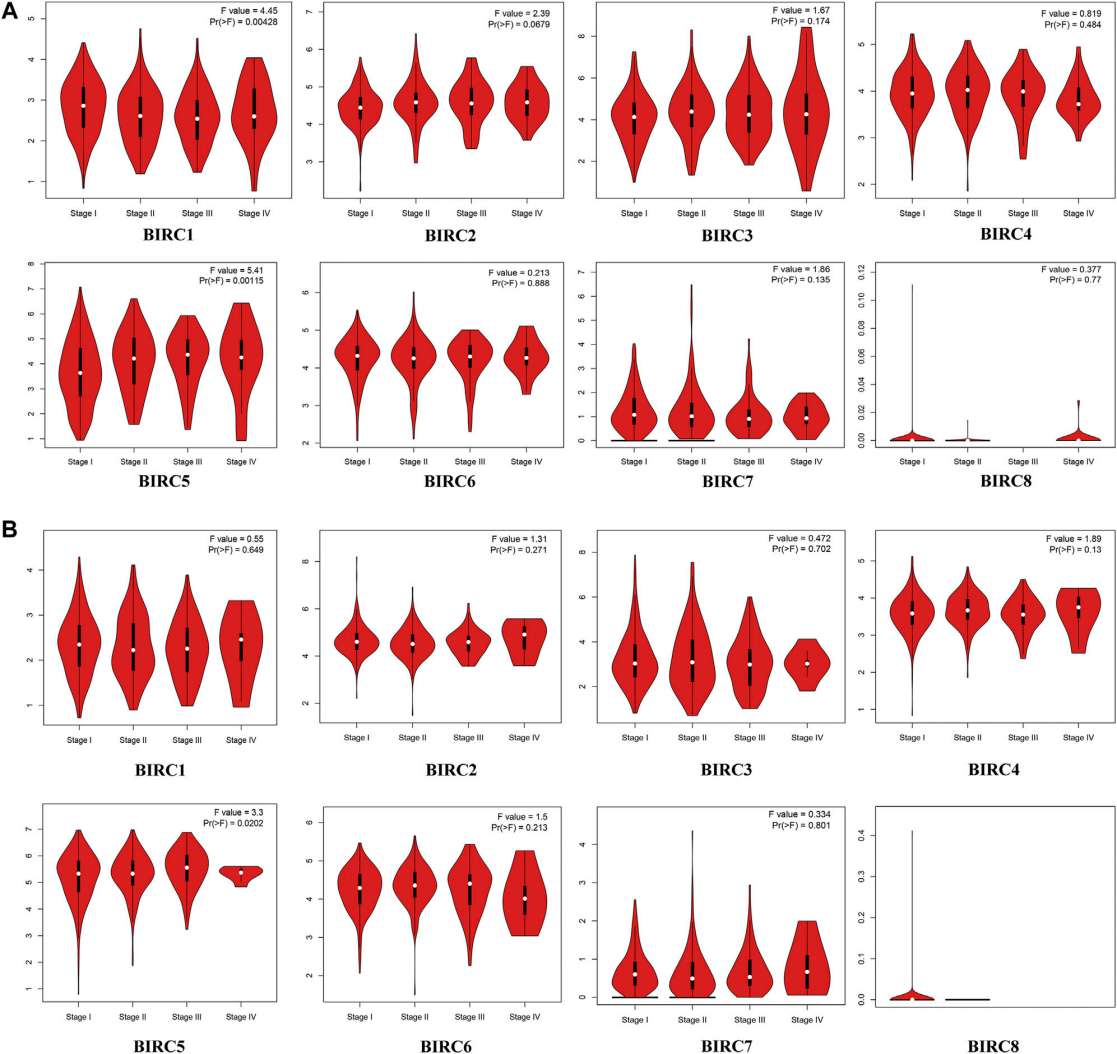
mRNA Expression of IAPs in Different Stages of NSCLC. **(A)** Correlation between mRNA expression of IAPs and tumor stage in LUAD patients. **(B)** Correlation between mRNA expression of IAPs and tumor stage in LUSC Patients. *Pr* < 0.05 indicates that the gene expression differs across stages. NSCLC, non–small cell lung cancer; LUAD, lung adenocarcinoma; LUSC, lung squamous cell carcinoma.

In the GEPIA dataset, *BIRC5* expression levels in LUAD and LUSC tissues were significantly higher than those in normal tissues, whereas the expression level of *BIRC1* was significantly lower in LUSC tissues than in the normal tissues (Figure 1). The expression of *BIRC1* and *BIRC5* significantly varied across LUAD stages, and the expression of *BIRC5* significantly varied across LUSC stages (Figure 2), suggesting that these IAPs may serve as potential biomarkers for diagnosis and cancer staging in NSCLC patients.

### IAPs are Associated with the Prognosis of Patients with NSCLC

**FIGURE 3:**
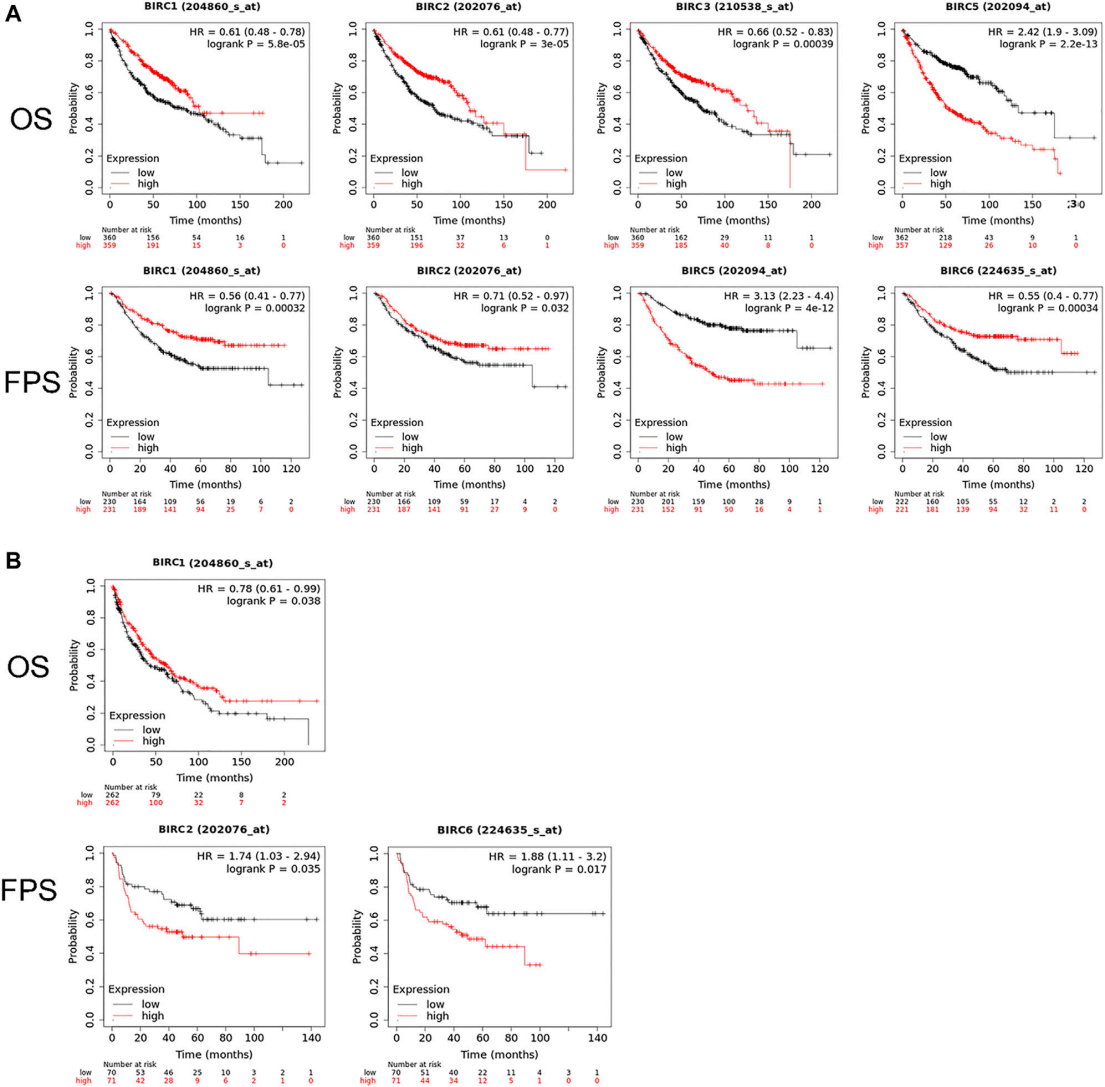
Prognostic value of IAP mRNA expression in patients with NSCLC. **(A)** IAPs significantly associated with the prognosis of LUAD patients. **(B)** IAPs significantly associated with the prognosis of LUSC patients. NSCLC, non–small cell lung cancer; LUAD, lung adenocarcinoma; LUSC, lung squamous cell carcinoma; OS, overall survival; PFS, progression-free survival.

The Kaplan–Meier curve and associated statistical analyses revealed that decreased *BIRC1*–*3* mRNA levels and increased *BIRC5* mRNA levels were significantly associated with the OS, whereas the decreased *BIRC1-2* and *6* mRNA levels and the increased *BIRC5* mRNA levels were significantly associated with the PFS of patients with LUAD (Table 2; Figure 3A). A decreased *BIRC1* mRNA level was significantly associated with OS, whereas increased *BIRC2* and *BIRC6* mRNA levels were significantly associated with the PFS of the patients with LUSC (Table 2; Figure 3B).

**TABLE 2:** Correlation of IAPs mRNA expression and prognosis in NSCLC by Kaplan–Meier plotter.

IAPs name (LUAD*)	Overall survival		Progression-free survival		IAPs name (LUSC*)	Overall survival		Progression-free survival	
	HR	*p* value	HR	*p* value		HR	*p* value	HR	*p* value
BIRC1	0.61(0.48–0.78)	**5.8E-05**	0.56(0.41–0.77)	**0.00300**	BIRC1	0.78(0.61–0.99)	**0.0375**	0.81(0.48–1.35)	0.4167
BIRC2	0.61(0.48–0.77)	**0.00003**	0.71(0.52–0.97)	**0.03220**	BIRC2	1.08(0.85–1.37)	0.5178	1.74(1.03–2.94)	**0.0353**
BIRC3	0.66(0.52–0.83)	**0.00039**	0.73(0.53–1.00)	0.04870	BIRC3	1.08(0.85–1.37)	0.5111	0.94(0.56–1.56)	0.8036
BIRC4	1.00(0.79–1.26)	0.99730	1.33(0.97–1.82)	0.07180	BIRC4	0.91(0.72–1.15)	0.4217	1.26(0.75–2.10)	0.3801
BIRC5	2.42(1.90–3.09)	**2.2E-13**	3.13(2.23–4.40)	**4.0E-12**	BIRC5	0.99(0.78–1.25)	0.9072	0.98(0.59–1.64)	0.9436
BIRC6	0.87(0.68–1.10)	0.24910	0.55(0.40–0.77)	**0.00030**	BIRC6	1.36(1.00–1.86)	0.0504	1.88(1.11–3.20)	**0.0172**
BIRC7	1.23(0.97–1.55)	0.08360	0.89(0.65–1.22)	0.48775	BIRC7	1.01(0.79–1.28)	0.9608	1.09(0.65–1.82)	0.7470

*LUAD, lung adenocarcinoma; LUSC, lung squamous cell carcinoma.

Bold values indicate *p* < 0.05.

### PPI Network

**FIGURE 4:**
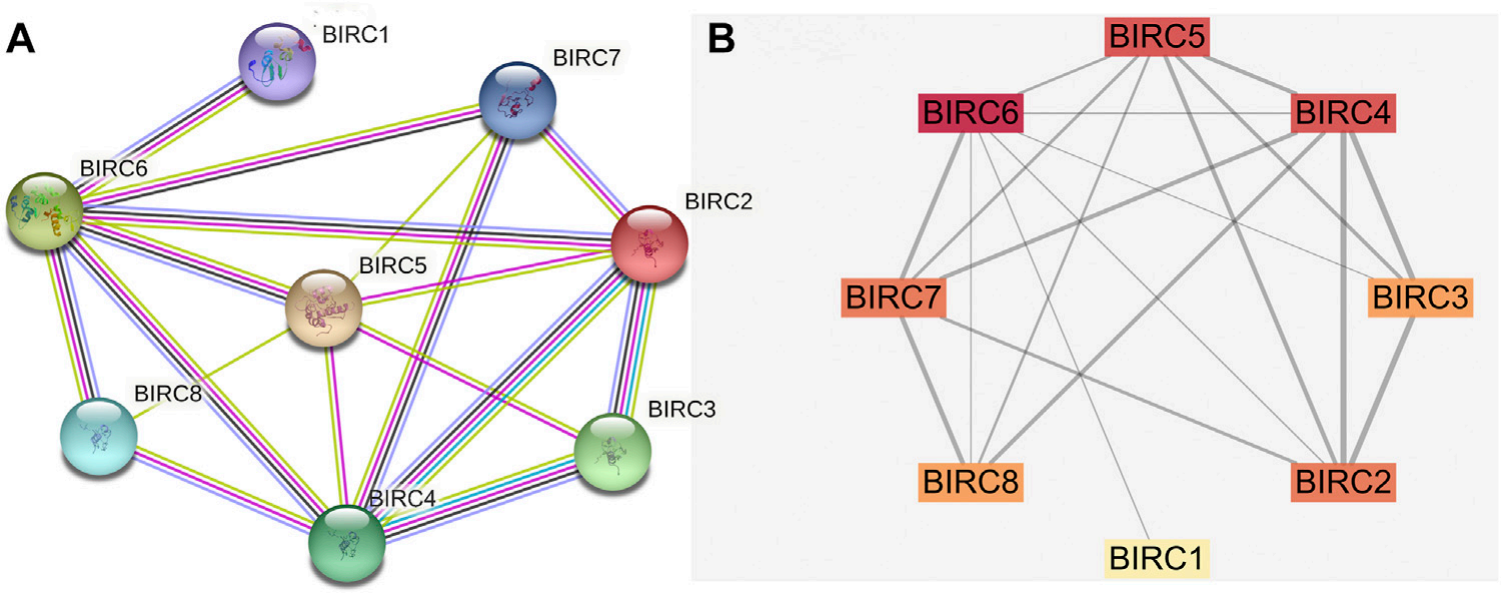
Protein-protein interaction network of IAPs. **(A)** STRING analysis. Different colors of lines indicate a different source of evidence: light blue, curated databases; rose, experimentally determined; green, gene neighborhood; red, gene fusions; dark blue, gene co-occurrence; light green, text mining; black, co-expression; purple, protein homology. **(B)** Cytoscape analysis. The darker the color, the greater the degree; the wider the line, the stronger the interaction.

The PPI network indicated that BIRC1 is co-expressed with BIRC6; BIRC2 is co-expressed with BIRC3, BIRC4, and BIRC6; BIRC3 is co-expressed with BIRC4; BIRC4 is co-expressed with BIRC6 and 7; BIRC5 is co-expressed with BIRC6; and BIRC6 is co-expressed with BIRC7 and 8 (Figure 4A). The interactions among these IAPs (except for BIRC5 with BIRC7 and BIRC8) have been experimentally validated. Overall, eight nodes formed a network of interactions with 19 edges (Figure 4B). The degree was greater than 4.75 for five nodes (average score): BIRC6, BIRC4, BIRC5, BIRC7, and BIRC2, from the highest to lowest (Supplementary Table S1). The combined score for 10 edges was greater than 0.686 (average score). The highest combined score was 0.971 based on the interaction of BIRC2 with BIRC4, followed by 0.943 (BIRC3 with BIRC4), 0.937 (BIRC2 with BIRC3), 0.827 (BIRC7 with BIRC8), 0.819 (BIRC6 with BIRC7), 0.779 (BIRC7 with BIRC4), 0.777 (BIRC2 with BIRC5), 0.771 (BIRC8 with BIRC4), 0.751 (BIRC5 with BIRC4), and 0.718 (BIRC3 with BIRC5) (Supplementary Table S2).

### Correlations Among IAPs in NSCLC

**FIGURE 5:**
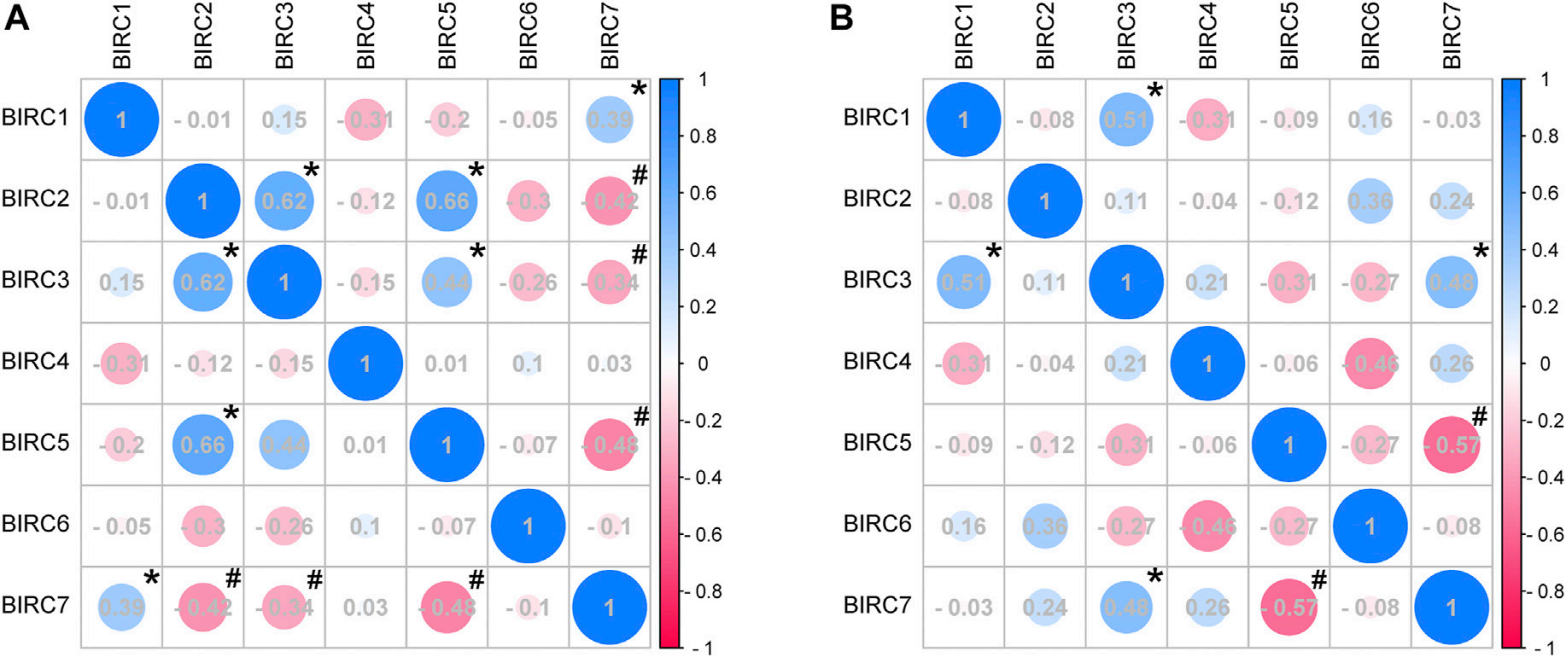
Correlation analysis of mRNA expression of IAPs in NSCLC. **(A)** Correlation analysis of IAPs expression in LUAD. **(B)** Correlation analysis of IAPs expression in LUSC. NSCLC, non–small cell lung cancer; LUAD, lung adenocarcinoma; LUSC, lung squamous cell carcinoma. Pink indicates a negative correlation and blue indicates a positive correlation; the darker the color, the large the Pearson correlation coefficient. * indicates a significantly positive relationship (*p* < 0.05); # indicates a significantly negative relationship (*p* < 0.05).

In LUAD, *BIRC1* was positively correlated with *BIRC7*, *BIRC2* was positively correlated with *BIRC3* and *BIRC5*, and *BIRC3* was positively correlated with *BIRC5*. Significant and negative correlations were identified between the following IAPs in LUAD: *BIRC2* with *BIRC7*, *BIRC3* with *BIRC7*, and *BIRC5* with *BIRC7* (Figure 5A and Table 3). In LUSC, *BIRC1* was positively correlated with *BIRC3* and *BIRC3* was also positively correlated with *BIRC7*, whereas *BIRC5* was negatively correlated with *BIRC7* (Figure 5B and Table 4).

**TABLE 3:** IAPs correlations in lung adenocarcinoma.

Gene name	Statistical indicator	BIRC1	BIRC2	BIRC3	BIRC4	BIRC5	BIRC6	BIRC7
BIRC1	Pearson	1	−0.009	0.149	−0.306	−0.195	−0.05	**0.388**
	Sig		0.954	0.36	0.055	0.228	0.761	**0.013**
	N	40	40	40	40	40	40	**40**
BIRC2	Pearson	−0.009	1	**0.617**	−0.118	**0.659**	−0.3	**−0.416**
	Sig	0.954		**0**	0.47	**0**	0.06	**0.008**
	N	40	40	**40**	40	**40**	40	**40**
BIRC3	Pearson	0.149	**0.617**	1	−0.15	**0.44**	−0.257	**−0.343**
	Sig	0.36	**0**		0.356	**0.005**	0.109	**0.03**
	N	40	**40**	40	40	**40**	40	**40**
BIRC4	Pearson	−0.306	−0.118	−0.15	1	0.008	0.095	0.034
	Sig	0.055	0.47	0.356		0.96	0.559	0.835
	N	40	40	40	40	40	40	40
BIRC5	Pearson	−0.195	**0.659**	**0.44**	0.008	1	−0.072	**−0.475**
	Sig	0.228	**0**	**0.005**	0.96		0.659	**0.002**
	N	40	**40**	**40**	40	40	40	**40**
BIRC6	Pearson	−0.05	−0.3	−0.257	0.095	−0.072	1	−0.105
	Sig	0.761	0.06	0.109	0.559	0.659		0.52
	N	40	40	40	40	40	40	40
BIRC7	Pearson	**0.388**	**−0.416**	**−0.343**	0.034	−**0.475**	−0.105	1
	Sig	**0.013**	**0.008**	**0.03**	0.835	**0.002**	0.52	
	N	**40**	**40**	**40**	40	**40**	40	40

Bold values indicate *p* < 0.05.

**TABLE 4:** IAPs correlations in lung squamous cell carcinoma.

Gene name	Statistical indicator	BIRC1	BIRC2	BIRC3	BIRC4	BIRC5	BIRC6	BIRC7
BIRC1	Pearson	1	−0.083	**0.51**	−0.314	−0.088	0.16	−0.027
	sig		0.742	**0.031**	0.204	0.729	0.525	0.915
	N	18	18	**18**	18	18	18	18
BIRC2	Pearson	−0.083	1	0.109	−0.04	−0.12	0.357	0.238
	sig	0.742		0.667	0.875	0.637	0.146	0.342
	N	18	18	18	18	18	18	18
BIRC3	Pearson	**0.51**	0.109	1	0.211	−0.307	−0.274	**0.48**
	sig	**0.031**	0.667		0.402	0.216	0.271	**0.044**
	N	**18**	18	18	18	18	18	**18**
BIRC4	Pearson	−0.314	−0.04	0.211	1	−0.055	−0.46	0.26
	sig	0.204	0.875	0.402		0.829	0.055	0.297
	N	18	18	18	18	18	18	18
BIRC5	Pearson	−0.088	−0.12	−0.307	−0.055	1	−0.267	**−0.568**
	sig	0.729	0.637	0.216	0.829		0.284	**0.014**
	N	18	18	18	18	18	18	**18**
BIRC6	Pearson	0.16	0.357	−0.274	−0.46	−0.267	1	−0.079
	sig	0.525	0.146	0.271	0.055	0.284		0.754
	N	18	18	18	18	18	18	18
BIRC7	Pearson	−0.027	0.238	**0.48**	0.26	**−0.568**	−0.079	1
	sig	0.915	0.342	**0.044**	0.297	**0.014**	0.754	
	N	18	18	**18**	18	**18**	18	18

Bold values indicate *p* < 0.05.

### IAPs Play Roles in Apoptosis and Ubiquitination in NSCLC

**FIGURE 6:**
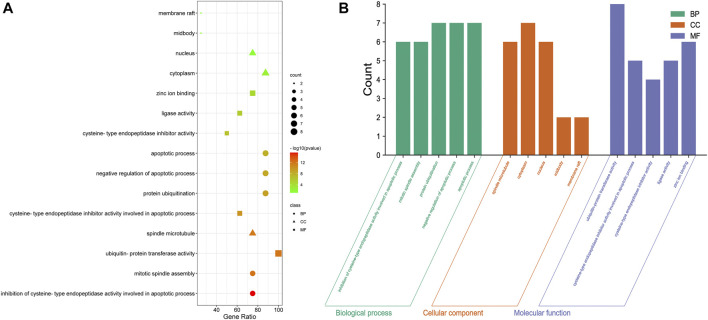
GO analysis of IAPs. **(A)** Enrichment dot bubble diagram. **(B)** Enrichment histogram. GO, Gene Ontology; BP, biological processes; CC, cellular components; MF, molecular functions.

GO enrichment analysis (Figure 6A, Supplementary Table S3) showed that the IAPs in NSCLC were significantly enriched in the biological process terms inhibition of cysteine-type endopeptidase activity involved in apoptotic process, mitotic spindle assembly, protein ubiquitination, negative regulation of apoptotic process, and apoptotic process terms; in the cellular component terms spindle microtubule, cytoplasm, nucleus, midbody, and membrane raft; and in the molecular function terms ubiquitin-protein transferase activity, cysteine-type endopeptidase inhibitor activity involved in apoptotic process, cysteine-type endopeptidase inhibitor activity, ligase activity, and zinc ion binding. More than half of the IAP members mainly participate in ubiquitin-protein transferase activity, protein ubiquitination, negative regulation of apoptotic process, apoptotic process, cytoplasm, and inhibition of cysteine-type endopeptidase activity involved in apoptotic process, mitotic spindle assembly, spindle microtubule, nucleus, and zinc ion binding (Figure 6B).

**FIGURE 7:**
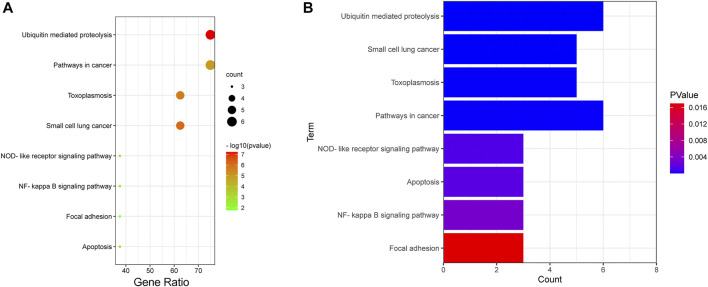
KEGG analysis of IAPs. **(A)** Enrichment dot bubble diagram. **(B)** Enrichment histogram. KEGG: Kyoto Encyclopedia of Genes and Genomes.

KEGG pathway analysis (Figure 7 and Supplementary Table S4) showed that the IAPs were most significantly enriched in ubiquitin-mediated proteolysis (*p* = 5.93E-08), followed by small cell lung cancer, toxoplasmosis, pathways in cancer, NOD-like receptor signaling pathway, NF-κB signaling pathway, focal adhesion, and apoptosis. More than half of the IAP members are mainly involved in the top four pathways (Figure 7B).

## Discussion

Although the role of IAPs in tumor development and progression has been partially confirmed in NSCLC, further bioinformatics analysis has yet to be performed (Rashed et al., 2019; Frazzi, 2021). This is the first study to comprehensively explore the transcriptional profiles, prognostic values, interactions, and functional enrichment of IAPs in different subtypes of NSCLC and across different tumor stages. Our findings can provide guidance for the development of IAPs as markers in the prevention, treatment, and prognosis for patients with NSCLC.

There has been no evidence of a role of *BIRC1* in NSCLC until now, and little has been reported of any association of *BIRC1* in cancer. A PTV-loaded nanocarrier was developed to trigger the apoptosis of glioblastoma multiforme cells by reducing the mRNA levels of *NFKB*, *IL6*, *BIRC1*, and *BIRC5* (Psc et al., 2021). Mig-6 exerts a tumor-suppressor function in murine endometrial cancer through downregulation of *BIRC1* expression (Kim et al., 2019). In our study, the GEPIA dataset revealed that the expression of *BIRC1* was lower in LUSC than that in normal tissues. Moreover, *BIRC1* mRNA expression was significantly different at least between two stages of LUAD and was associated with OS or PFS in patients with NSCLC. These data suggest that downregulation of *BIRC1* possibly plays a tumor-suppressor function in NSCLC development.

A previous study showed that *BIRC2* expression regulates the apoptosis and survival of NSCLC cells: downregulating *BIRC2* expression indirectly induces NSCLC cell apoptosis by preventing formation of the caspase-8–activating platform (Yang and Wang, 2016; Jian et al., 2019). The overexpression of *BIRC2*, regulated by Pellino-1, contributes to the oncogenesis of A549 and H1299 cells, which are both LUAD cell lines, and promotes cancer cell survival (Jeon et al., 2016; Xv et al., 2021). Consistently, we found that downregulated *BIRC2* expression was associated with the prolonged survival time of patients with LUAD.

In our study, only *BIRC3* expression was positively correlated with the OS of patients with LUAD. There is substantial evidence pointing to the pro-survival and anti-apoptotic roles of BIRC3 in cancer cells; however, not all data are consistent (Frazzi, 2021). An *in vitro* study showed that RNA-binding motif 10 overexpression inhibited the malignant behaviors of A549 and H1299 cells by inducing the expression of AKT2, BIRC3, and JUN (Guan et al., 2017). However, Dubois et al. (2019) reported that overexpression of *BIRC3* regulated by *RASSF1A* depletion decreased the rate of cancer cell apoptosis. Similarly, upregulation of *BIRC3* expression *via* Pellino-1 overexpression in A549 and H1299 cells promoted lung oncogenesis and survival, and *BIRC3* also demonstrated a strong positive correlation with Pellino-1 in human LUAD tissues (Yang and Wang, 2016). Therefore, the mechanism of BIRC3 in cancer needs further study.

Surprisingly, we did not identify a specific role of *BIRC4* in the patients with LUAD or LUSC on the basis of the databases analyzed in this study. However, several *in vitro* studies have suggested an anti-NSCLC role of *BIRC4*. Hydrogen gas was suggested to promote the apoptosis of A549 cells by reducing the expression of *BIRC4* (Zhang et al., 2020a). Combined with other drugs in treating NSCLC *in vitro*, TRAIL induced cell apoptosis by inhibiting *BIRC4* expression and increasing cytotoxicity (Deok et al., 2018; Kim et al., 2019). Moreover, the positive rate of *BIRC4* mRNA expression in the pathological tissues of NSCLC patients was significantly higher than that in paracancerous tissues (De-Xuan et al., 2017). Although the expression of BIRC4 varies *in vitro* and *in vivo*, further *in vitro* experiments can represent an important starting point to better understand its regulation mechanisms and functions *in vivo*.

Unlike other IAPs, *BIRC5* is strongly expressed in most tumors but is not expressed or is expressed at only low levels in most normal differentiated tissues (Xiao and Li, 2015; Mazur et al., 2018). Consistently, we found that *BIRC5* expression levels were significantly higher in tumor tissues than in normal tissues. Previous studies have suggested *BIRC5* as a predictive biomarker in NSCLC, especially for LUAD (Zhang et al., 2020b; Haakensen et al., 2020). In addition, in the present study, BIRC5 emerged as the most significant IAP that could be developed as a marker for preventing and treating NSCLC patients. Low expression of *BIRC5* mRNA was also previously positively correlated with NSCLC patient survival (Cao et al., 2019; Nitschkowski et al., 2019; Rashed et al., 2019; Zhang et al., 2020b).

*BIRC6* has been suggested as a progression marker in NSCLC (Dong et al., 2013; Gharabaghi and Asadi, 2016), which was also associated with the PFS of the patients with NSCLC in our study. However, previous studies did not distinguish among different subtypes of NSCLC. Here, we show that *BIRC6* expression actually shows an opposite association with prognosis in patients with LUAD and LUSC: Increased *BIRC6* expression was significantly associated with the PFS of patients with LUAD, whereas decreased *BIRC6* was significantly associated with the PFS of patients with LUSC. This suggests that *BIRC6* is a potential biomarker for differentiating different types of NSCLC. No specific roles of *BIRC7* and *BIRC8* in NSCLC were identified in this study or in the literature to date.

From GO and KEGG enrichment analysis, we found that all eight members of the IAP family are enriched in ubiquitin-protein transferase activity, and most of them (six of eight) are enriched in ubiquitin-mediated proteolysis. The ubiquitin–proteasome system has become a key system of pathogenesis in several cancers (Senft et al., 2018). Thevebioside (an active ingredient from Traditional Chinese Medicine) was reported to inhibit the tumor growth of NSCLC through inhibiting SRC-3–mediated IGF-1R–PI3K-AKT signaling *via* ubiquitination to induce cellular apoptosis (Yao et al., 2020). In addition, deregulation of APC/C (a representative E3 ligase) together with its co-activators cell division cycle 20 (CDC20) or CDC20-like protein 1 (CDH1) has been associated with cancers (Jeon et al., 2016). Overexpression of Pellino-1 (an E3 ubiquitin ligase) is dependent on the expression of BIRC3 in human lung cancer cells, resulting in increased cell survival and colony forming ability (Jeon et al., 2016). SKP2 promotes programmed cell death protein 4 degradation through phosphorylation and ubiquitination, resulting in increased proliferation and radiation tolerance of breast cancer cells (Li et al., 2019). In addition, IAPs were also found to play a role in the NOD-like receptor signaling pathway and NF-κB signaling pathway (Mann and Oakley, 2005; Liu et al., 2019; Kumar et al., 2021). Thus, we speculate that the dysregulation of IAPs has more effective role in the inflammatory response.

## Conclusion

In this study, we systematically analyzed the expression and prognostic value of IAPs in different subtypes of NSCLC, which can help to provide a more thorough understanding of the molecular biological properties of this cancer. Our results indicate that *BIRC1* and *BIRC5* are potential diagnostic markers for both LUAD and LUAC. *BIRC1*, *BIRC2*, and *BIRC5* are potential prognostic markers for LUAD, whereas *BIRC2* and *BIRC6* are prognostic markers for patients with NSCLC. From GO and KEGG enrichment analysis, we found that most IAP members are associated with ubiquitin and apoptosis. These highlight new targets for the early detection, treatment, and management of NSCLC.

## Data Availability

The original contributions presented in the study are included in the article/Supplementary Material; further inquiries can be directed to the corresponding author.

## Supplementary Material

Supplementary Table S1 The degree of IAPs from PPI network.

Supplementary Table S2 The combined score of IAPs from PPI network.

Supplementary Table S3 Go Function enrichment analysis of IAPs.

Supplementary Table S4 KEGG pathway enrichment.

## References

[B1] Cao Y. Zhu W. Chen W. Wu J. Hou G. Li Y. (2019). Prognostic Value of BIRC5 in Lung Adenocarcinoma Lacking EGFR, KRAS, and ALK Mutations by Integrated Bioinformatics Analysis. Dis. markers 2019 (3), 1–12. 10.1155/2019/5451290 PMC6481100 31093306

[B2] Chen J. Huang X. Tao C. Xiao T. Li X. Zeng Q. (2019). Artemether Attenuates the Progression of Non-small Cell Lung Cancer by Inducing Apoptosis, Cell Cycle Arrest and Promoting Cellular Senescence. Biol. Pharm. Bull. 42 (10), 1720–1725. 10.1248/bpb.b19-00391 31378747

[B3] De-Xuan L. I. Ya-Shan L. I. Wang J. Zhang B. J. Hong Y. Zhang Q. Y. (2017). Expression and Clinical Significance of Survivin, Skp2 and XIAP mRNA in Non-small Cell Lung Cancer. Prog. Mod. Biomed. 17 (31), 6159–6162. 10.13241/j.cnki.pmb.2017.31.037

[B4] Deok A. Hyo L. Jisung H. Han H. Bonglee K. Bumsang S. (2018). Lambertianic Acid Sensitizes Non-small Cell Lung Cancers to TRAIL-Induced Apoptosis via Inhibition of XIAP/NF-κB and Activation of Caspases and Death Receptor 4. Int. J. Mol. ences 19 (5), 1476. 10.3390/ijms19051476 PMC5983579 29772677

[B5] Dong X. Lin D. Low C. Vucic E. A. English J. C. Yee J. (2013). Elevated Expression of BIRC6 Protein in Non-small-cell Lung Cancers Is Associated with Cancer Recurrence and Chemoresistance. J. Thorac. Oncol. 8 (2), 161–170. 10.1097/jto.0b013e31827d5237 23287853

[B6] Dubois F. Keller M. Hoflack J. Maille E. Antoine M. Westeel V. (2019). Role of the YAP-1 Transcriptional Target cIAP2 in the Differential Susceptibility to Chemotherapy of Non-small-cell Lung Cancer (NSCLC) Patients with Tumor RASSF1A Gene Methylation from the Phase 3 IFCT-0002 Trial. Cancers 11 (12), 1835. 10.3390/cancers11121835 PMC6966477 31766357

[B7] Frazzi R. (2021). BIRC3 and BIRC5: Multi‐faceted Inhibitors in Cancer. Cell Biosci. 11 (1), 8. 10.1186/s13578-020-00521-0 33413657 PMC7792207

[B8] Gharabaghi M. A. Asadi M. (2016). Diagnostic Investigation of BIRC6 and SIRT1 Protein Expression Level as Potential Prognostic Biomarkers in Patients with Non-small Cell Lung Cancer. Clin. Respir. J. 12 (2), 633–638. 10.1111/crj.12572 27768839

[B9] Guan G. Li R. Tang W. Liu T. Su Z. Wang Y. (2017). Expression of RNA-Binding Motif 10 Is Associated with Advanced Tumor Stage and Malignant Behaviors of Lung Adenocarcinoma Cancer Cells. Tumour Biol. 39 (3), 1010428317691740. 10.1177/1010428317691740 28347232

[B10] Haakensen V. D. Khadse A. Sandhu V. Halvorsen A. R. Solberg S. K. Jørgensen L. H. (2020). Molecular Characterisation of TP53 Mutated Squamous Cell Carcinomas of the Lung to Identify Putative Targets for Therapy. Int. J. Cancer 147 (10), 2957–2966. 10.1002/ijc.33121 32468587 PMC7540694

[B11] Jeon Y. K. Kim C. K. Koh J. Chung D. H. Ha G.-H. (2016). Pellino-1 Confers Chemoresistance in Lung Cancer Cells by Upregulating cIAP2 through Lys63-Mediated Polyubiquitination. Oncotarget 7 (27), 41811–41824. 10.18632/oncotarget.9619 27248820 PMC5173098

[B12] Ji J. Yu Y. Li Z.-L. Chen M.-Y. Deng R. Huang X. (2018). XIAP Limits Autophagic Degradation of Sox2 and Is A Therapeutic Target in Nasopharyngeal Carcinoma Stem Cells. Theranostics 8 (6), 1494–1510. 10.7150/thno.21717 29556337 PMC5858163

[B13] Khan S. A. Burke M. Zhu F. Yang D.-H. Dubyk C. Mehra R. (2021). Survivin Expression and Impact on Head and Neck Cancer Outcomes. Oral Oncol. 112, 105049. 10.1016/j.oraloncology.2020.105049 33221541 PMC10916757

[B14] Kim Y. H. Shin E. A. Jung J. H. Park J. E. Koo J. Koo J. I. (2019). Galbanic Acid Potentiates TRAIL Induced Apoptosis in Resistant Non-small Cell Lung Cancer Cells via Inhibition of MDR1 and Activation of Caspases and DR5. Eur. J. Pharmacol. 847, 91–96. 10.1016/j.ejphar.2019.01.028 30689998

[B15] Kumar S. Nandi A. Singh S. Regulapati R. Li N. Tobias J. W. (2021). Dll1+ Quiescent Tumor Stem Cells Drive Chemoresistance in Breast Cancer through NF-Κb Survival Pathway. Nat. Commun. 12 (1), 432. 10.1038/s41467-020-20664-5 33462238 PMC7813834

[B16] Li C. Du L. Ren Y. Liu X. Jiao Q. Cui D. (2019). SKP2 Promotes Breast Cancer Tumorigenesis and Radiation Tolerance through PDCD4 Ubiquitination. J. Exp. Clin. Cancer Res. 38 (1), 76. 10.1186/s13046-019-1069-3 30760284 PMC6375223

[B17] Liu B. Ricarte Filho J. Mallisetty A. Villani C. Kottorou A. Rodgers K. (2020). Detection of Promoter DNA Methylation in Urine and Plasma Aids the Detection of Non-small Cell Lung Cancer. Clin. Cancer Res. 26 (16), 4339–4348. 10.1158/1078-0432.ccr-19-2896 32430478 PMC7442601

[B18] Liu P. Lu Z. Liu L. Li R. Liang Z. Shen M. (2019). NOD-like Receptor Signaling in Inflammation-Associated Cancers: From Functions to Targeted Therapies. Phytomedicine 64, 152925. 10.1016/j.phymed.2019.152925 31465982

[B19] Mann D. A. Oakley F. (2005). NF-κB: a Signal for Cancer. J. Hepatol. 42 (4), 610–611. 10.1016/j.jhep.2005.01.007 16715553

[B20] Mazur J. Roy K. Kanwar J. R. (2018). Recent Advances in Nanomedicine and Survivin Targeting in Brain Cancers. Nanomedicine 13 (1), 105–137. 10.2217/nnm-2017-0286 29161215

[B21] Nitschkowski D. Marwitz S. Kotanidou S. A. Reck M. Kugler C. Rabe K. F. (2019). Live and let die: epigenetic modifications of Survivin and Regucalcin in non-small cell lung cancer tissues contribute to malignancy. Clin. Epigenet 11 (1), 157. 10.1186/s13148-019-0770-6 PMC6852724 31718698

[B22] Psc A. Mk B. Amc C. As B. Dda D. (2021). Multifunctional Silica-Coated Mixed Polymeric Micelles for Integrin-Targeted Therapy of Pediatric Patient-Derived Glioblastoma. Mater. Sci. Eng. C 128, 112261. 10.1016/j.msec.2021.112261 34474820

[B23] Rashed R. A. Rahouma M. Abo Elfetouh R. Aziz H. Kamel M. K. (2019). Effect of Serum Survivin on Survival Among Non-small Cell Lung Cancer Patients: NCI Experience. Ann. Oncol. 30, v51. 10.1093/annonc/mdz239.072

[B24] Senft D. Qi J. Ronai Z. e. A. (2018). Ubiquitin Ligases in Oncogenic Transformation and Cancer Therapy. Nat. Rev. Cancer 18 (2), 69–88. 10.1038/nrc.2017.105 29242641 PMC6054770

[B25] Siegel R. L. Miller K. D. Jemal A. (2020). Cancer Statistics, 2020. CA A. Cancer J. Clin. 70 (1), 7–30. 10.3322/caac.21590 31912902

[B26] Upadhya A. Yadav K. S. Misra A. (2021). Targeted Drug Therapy in Non-small Cell Lung Cancer: Clinical Significance and Possible Solutions-Part I. Expert Opin. Drug Deliv. 18 (1), 73–102. 10.1080/17425247.2021.1825377 32954834

[B27] Xiao M. Li W. (2015). Recent Advances on Small-Molecule Survivin Inhibitors. Curr. Med. Chem. 10.2174/0929867322666150114102146 PMC5342940 25613234

[B28] Xv F. Liu W. Chen Y. Fang H. Zuo J. (2021). miR-623 Targets DNM2 to Regulate the Apoptosis of Non-small Cell Lung Cancer Cell A549. Int. J. Respiration 41 (10), 771–776. Available at https://kns.cnki.net/kcms/detail/detail.aspx?dbcode=CJFD&dbname=CJFDZHYX&filename=GWHX202110009&uniplatform=NZKPT&v=HnAahxUC1PVMULeG3ei6y8TMPsoZqOr2vmEqExwGNL1kGi_5pQWuDYbgyXowxzuk

[B29] Yang C. Wang H. (2016). LCL161 Increases Paclitaxel-Induced Apoptosis by Degrading cIAP1 and cIAP2 in NSCLC. J. Exp. Clin. Cancer Res. CR 35 (1), 158. 10.1186/s13046-016-0435-7 27737687 PMC5062899

[B30] Yao C. Su L. Zhang F. Zhu X. Xu Z. (2020). Thevebioside, the Active Ingredient of Traditional Chinese Medicine, Promotes Ubiquitin-Mediated SRC-3 Degradation to Induce NSCLC Cells Apoptosis. Cancer Lett. 493, 167–177. 10.1016/j.canlet.2020.08.011 32829007

[B31] Zhang Y. Chen G. Yan F. Z. Wang F. L. Wang C. D. (2020a). Mechanism of Hydrogen Gas Promoted Apoptosis of Lung Adenocarcinoma A549 Cells through XIAP and BIRC3. Preprint. 10.21203/rs.3.rs-111317/v1 36204886

[B32] Zhang Y. Sun Y. Jia Y. Zhang Q. Zhu P. Ma X. (2020b). α5-nAChR and Survivin: Two Potential Biological Targets in Lung Adenocarcinoma. J. Cell Physiol. 236 (3), 1787–1797. 10.1002/jcp.29956 33196129

