# Supplementary material for: Comprehensive Analysis of Inhibitor of Apoptosis Protein Expression and Prognostic Significance in Non–Small Cell Lung Cancer
Source: Front Genet. 2021 Dec 2;12:764270. doi: 10.3389/fgene.2021.764270 (PMC8675358; doi:10.3389/fgene.2021.764270)
Supplement: Supplementary file 1 [file DataSheet1.docx]

**Table S1 The degree of IAPs from PPI network**

| No. | Gene name | Degree |
| --- | --- | --- |
| 1 | BIRC6 | 7 |
| 2 | BIRC4 | 6 |
| 3 | BIRC5 | 6 |
| 4 | BIRC7 | 5 |
| 5 | BIRC2 | 5 |
| 6 | BIRC8 | 4 |
| 7 | BIRC3 | 4 |
| 8 | BIRC1 | 1 |
| 9 | Average degree | 4.75 |

**Table S2 The combined score of IAPs from PPI network**

| No. | Gene name | Combined_score |
| --- | --- | --- |
| 1 | BIRC2 (interacts with) BIRC4 | 0.971 |
| 2 | BIRC3 (interacts with) BIRC4 | 0.943 |
| 3 | BIRC2 (interacts with) BIRC3 | 0.937 |
| 4 | BIRC7 (interacts with) BIRC8 | 0.827 |
| 5 | BIRC6 (interacts with) BIRC7 | 0.819 |
| 6 | BIRC7 (interacts with) BIRC4 | 0.779 |
| 7 | BIRC2 (interacts with) BIRC5 | 0.777 |
| 8 | BIRC8 (interacts with) BIRC4 | 0.771 |
| 9 | BIRC5 (interacts with) BIRC4 | 0.751 |
| 10 | BIRC3 (interacts with) BIRC5 | 0.718 |
| 11 | BIRC2 (interacts with) BIRC7 | 0.667 |
| 12 | BIRC5 (interacts with) BIRC7 | 0.646 |
| 13 | BIRC5 (interacts with) BIRC6 | 0.598 |
| 14 | BIRC5 (interacts with) BIRC8 | 0.585 |
| 15 | BIRC2 (interacts with) BIRC6 | 0.467 |
| 16 | BIRC6 (interacts with) NAIP | 0.461 |
| 17 | BIRC6 (interacts with) BIRC4 | 0.456 |
| 18 | BIRC6 (interacts with) BIRC8 | 0.434 |
| 19 | BIRC3 (interacts with) BIRC6 | 0.430 |
| 20 | Average score | 0.686 |

| Group | Term | Count | PValue | Gene ratio(%) |
| --- | --- | --- | --- | --- |
| Biological process | inhibition of cysteine-type endopeptidase activity involved in apoptotic process | 6 | 2.38E-16 | 75.0 |
| Biological process | mitotic spindle assembly | 6 | 7.10E-13 | 75.0 |
| Biological process | protein ubiquitination | 7 | 6.30E-10 | 87.5 |
| Biological process | negative regulation of apoptotic process | 7 | 2.62E-09 | 87.5 |
| Biological process | apoptotic process | 7 | 9.82E-09 | 87.5 |
| Cellular component | spindle microtubule | 6 | 1.36E-12 | 75.0 |
| Cellular component | cytoplasm | 7 | 0.002918 | 87.5 |
| Cellular component | nucleus | 6 | 0.027595 | 75.0 |
| Cellular component | midbody | 2 | 0.048518 | 25.0 |
| Cellular component | membrane raft | 2 | 0.076505 | 25.0 |
| Molecular function | ubiquitin-protein transferase activity | 8 | 1.00E-12 | 100.0 |
| Molecular function | cysteine-type endopeptidase inhibitor activity involved in apoptotic process | 5 | 9.14E-11 | 62.5 |
| Molecular function | cysteine-type endopeptidase inhibitor activity | 4 | 2.60E-07 | 50.0 |
| Molecular function | ligase activity | 5 | 2.16E-06 | 62.5 |
| Molecular function | zinc ion binding | 6 | 2.95E-05 | 75.0 |

**Table S3 Go Function enrichment analysis of IAPs**

**Table S4 KEGG pathway enrichment**

| Term | Count | PValue |
| --- | --- | --- |
| Ubiquitin mediated proteolysis | 6 | 5.93E-08 |
| Small cell lung cancer | 5 | 7.39E-07 |
| Toxoplasmosis | 5 | 2.09E-06 |
| Pathways in cancer | 6 | 1.13E-05 |
| NOD-like receptor signaling pathway | 3 | 0.001332 |
| Apoptosis | 3 | 0.00163 |
| NF-kappa B signaling pathway | 3 | 0.003187 |
| Focal adhesion | 3 | 0.01697 |
